# Supplementary material for: Treatment of HMG-CoA Lyase Deficiency—Longitudinal Data on Clinical and Nutritional Management of 10 Australian Cases
Source: Nutrients. 2023 Jan 19;15(3):531. doi: 10.3390/nu15030531 (PMC9920477; doi:10.3390/nu15030531)
Supplement: Supplementary file 1 [file nutrients-15-00531-s001.zip › nutrients-2109519-supplementary.pdf]

Treatment of HMG-CoA Lyase deficiency – Longitudinal data on clinical and nutritional management of ten Australian cases

Online Supplementary Material

Patient 1 (References: Faull et al., 1976 [2]; Wysocki et al., 1979 [8]; Wysocki et al., 1986 [9]; Shilkin et al., 1981 [10])

The first patient with this disorder was published by Faull et al in 1976. The male infant was well until seven months of age, when he developed drowsiness as well as episodes of cyanosis and apnoea in the context of a gastroenteritic illness. The report indicated the presence of hypoglycaemia (blood glucose level 0.3 mmol/L; standard reference range 3.0-5.5) and metabolic acidosis (bicarbonate 11mmol/L; standard reference range 16 – 22). He had hepatomegaly without deranged liver function. Urine organic acids by gas-liquid chromatography mass spectrometry abnormally elevated 3-hydroxy-3-methylglutarate (3-HMG), as well as 3-hydroxyisovalerate (3-HIVA), 3-methylglutarate (3-MGL) and 3-methylglutaconate (3-MGC). It was identified that this pattern differed from the other defects of leucine catabolism known at the time (maple syrup urine disease, isovaleric acidaemia and 3-methylcrotonyl-CoA carboxylase deficiency), and was likely due to a more distal blockade, in the conversion of 3HMG into acetoacetate and acetyl-CoA.

The patient was commenced on a moderately protein-restricted diet, predominantly based on carbohydrates. Episodes of decompensation were managed at home with additional glucose based on the above previously published literature. It is understood that he lived into adulthood, as the monitoring laboratory received samples until the age of 35 years. He is lost to clinical follow-up.

Patient 2 (References: Hammond et al, 1984 [11]; Jones et al. 1997 [22]; Grunert et al., 2020 [7])

Patient 2 is of Chilean origin and was adopted at four weeks of age. She first presented at three months of age following a brief viral prodrome, with encephalopathy, hepatomegaly and seizures. She had metabolic acidosis and hypoketotic hypoglycaemia (exact values not available). Urine organic analysis demonstrated the typical pattern seen in HMGCL deficiency (Table 1). HMGCL enzyme activity, assayed in patient fibroblasts, was markedly reduced (1% of controls). She was diagnosed with severe sensorineural hearing loss at an early age, and this in combination with slowly progressive retinal degeneration, can be explained by a recent molecular diagnosis of Usher Syndrome.

She had a total of three further episodes of mild acidosis in her first year of life, and a few more admissions over the next four years without significant acidosis. Her management during this time included a low-protein diet (with the addition of MSUD formula during early infancy) and uncooked cornstarch overnight to prevent hypoglycaemia. She developed seizures at eight months of age, and seizure control improved commencement of carbamazepine. At age 3.5 years, a modified Gessell Developmental Assessment demonstrated mild-to-moderate global developmental delay, with a relative strength in gross motor skills (three years' equivalent) and a relative weakness in language (15 months' equivalent).

From age 5 to 13 years, she had two metabolic decompensations (with gastroenteritis and a wisdom tooth extraction respectively), both requiring intensive care admission. During each episode, she was managed with protein restriction, high concentrations of dextrose-containing fluids delivered intravenously and regular ondansetron. She had one further admission aged 28 years without metabolic decompensation, but has had no hospital admissions for 11 years.

She is now aged 39 years. She has a persistent mild intellectual disability. Her growth parameters are normal, and she has been seizure-free for over 10 years. She has osteoporosis. She has a self-restricted diet (consisting of bananas, white bread, peanut butter, honey and apple juice) which is slowly broadening in adulthood, She has restricted total energy intake of approximately 1250 Kcals/ day (although is quite sedentary.) This diet provides approximately 70% of her calories from carbohydrates, and 12% from protein and 18% fat.

<u>Patient 3</u>

Patient 3, the first child of non-consanguineous parents, was born by urgent Caesarian section at 42 weeks' gestation, due to fetal distress. APGAR scores were 1^1 and 8^5. Respiratory distress was noted after birth, with evidence of mild meconium aspiration on the chest radiograph. On day four, poor feeding was noted and the baby became inactive and hypotonic. Blood glucose level was 0.1 mmol/L (3.0 - 5.5) and arterial blood gas showed pH 7.27, bicarbonate of 10 mmol/L (18-24) with base excess -17. She was treated with recurrent doses of sodium bicarbonate. The acidosis persisted over the next two days. Urine organic acids showed a major peak of 3-MGC, and smaller peaks of 3-MGL, 3-HMG and 3-HIVA. Fibroblast enzyme activity of HMGCL was <0.4 units/g protein (4.6-6.7).

At age four years, she had viral meningitis with acidosis which required treatment with bicarbonate and glucose infusion. She had nine admissions in total up to age 13 years, and subsequently no hospital admissions for 20 years.

She was initially managed in childhood with supplemental maltodextrin and sodium bicarbonate; the latter was ceased at seven years of age. 100 mg/kg/day of L-Carnitine was commenced at eight years of age, increasing to a maximum of 1g twice daily in adult life. In her early teen years, maltodextrin was ceased and a formula containing both fat and maltodextrin (Duocal^{TM}: Nutricia – Utrecht, Netherlands) was commenced. This was discontinued in adult life.

She has maintained a low body weight throughout life (body mass index 17-18kg/m^2), and is now 33 years old. There have been no concerns regarding her cognition; she had average academic achievement at school, did not pursue higher degrees and works as an administrator.

<u>Patient 4</u> (References: Dalkeith et al., 2013 [20]; Bhattacharya et al., 2020 [21])

Patient 4 is the second male child of non-consanguineous Slovakian parents. He had an uneventful birth and infancy. He presented at approximately 10 months of age with metabolic acidosis (data not available) when cow's milk was introduced. Protein intake appeared to have increased from 13g/day to 25g/ day. HMGCLD was diagnosed on the basis of typical biochemistry including gross elevation of urinary 3-HMG. He was stabilised on a reduced protein (1.5-2g/kg/day) and reduced fat (25-30% of total energy) diet. He was lost to follow-up from age 15 months until 16 years. At this time, he had been attending mainstream schooling, with normal academic achievement.

At age 16 years, he presented to the local emergency department after two days of lethargy, disorientation and slurred speech. Baseline assessment noted that tachycardia, tachypnoea and hypothermia (34.7 degrees Celsius). His Glasgow Coma Scale (GCS) was 15. Initial biochemistry demonstrated pH 7.12, bicarbonate 6.8 mmol/L (18–24), lactate 16 mmol/L (0–2), base excess −20 (−2 to + 2) and anion gap 29 mmol/L (8–18), with a blood glucose of 2 mmol/L (3.0–5.5). Serum ammonia was elevated at 455 µmol/L (<50). He was resuscitated with IV fluids (10% dextrose and saline) but

developed fixed and dilated pupils; a transcranial bolt was applied to urgently reduce intracranial pressure.

He commenced on 600 mg/kg/day of sodium D,L 3-hydroxybutyrate (S-DL-3OHB). He began to recover and was discharged home 24 days after admission: however he sustained severe cortical blindness secondary to occipital lobe infarction, confirmed on a subsequent brain MRI scan. He has been stable for 11 years since this episode: now at age 27 years, despite being registered blind, he has normal intellect, mobility and tone.

<u>Patient 5</u> (Reference: Dalkeith et al., 2013 [20])

Patient 5, the first child of consanguineous Israeli parents, presented on day 3 of life with hypothermia, poor feeding and vomiting. Venous glucose was 1.4 mmol/L (3.0 – 5.5) and venous blood gas showed pH 7.21, bicarbonate of 8 mmol/L (18-24) and base excess -18mmol/L (-2 to +2), with a raised anion gap of 27mmol/L (8-18). Serum ammonia was 245 µmol/L (<100). He was empirically administered 1 mg of vitamin B12 and transferred to tertiary unit. He was managed with 10 % dextrose-containing fluids to maintain normal glucose levels. The biochemical diagnosis of HMGCL deficiency was confirmed on day 4 of life, at which point protein and fat were restricted by adding maltodextrin to feeds (100mL/kg expressed human milk with 50mL/kg maltodextrin, concentrated to meet 120% of his estimated energy requirement, EER).

Solids were introduced at five months, whilst breastfed, avoiding foods with more than 2g of fat per 100g. He had one serve of legumes at six months. Reviewing the dietary history at seven months of age, he was receiving 27% of his total energy from fat and only 8% from protein. Protein intake from food was increased from 12 months to be approximately 1g/kg, with fat restricted to foods containing <3g/100g. This plan was maintained until five years of age, when fat and protein were liberalised somewhat, with the aim of avoiding high fat and protein foods. Emergency management for illness includes frequent feeds of maltodextrin solution to meet 120% of his EER.

Brain MRI at six weeks of age demonstrated white matter changes; regular S-DL-3OHB was administered at 300 mg/kg/day in five divided doses thereafter. A repeat MRI scan at one year of age appeared to show improvement, with subsequent stabilization on the subsequent MRI at two years. At this point, 4hrly daytime dosing was discontinued and single night-time dosing commenced, and MRI appearances continued to be stable at seven years of age. Uncooked cornstarch 2g/kg/day at night was recommended at 1 year of age but compliance was never achieved.

Patient 5 has had three admissions to hospital over 13 years. He had one admission aged two years with oral herpes simplex infection leading to poor oral intake. This admission included four days where he was maintained on intravenous dextrose (delivering glucose at 7.7 mg/kg/minute), with S-DL-3OHB being administered at 900 mg/kg/day for 48 hours before enteral feeds were re-established. S-DL-3OHB dosing was concomitantly reduced. He had no evidence of metabolic acidosis, hypoglycemia nor hyperammonemia during this admission. He now takes 150 mg/kg/dose of S-DL-3OHB as a regular dose at night. He attends mainstream school with no support, having normal intellect. He is able to exercise normally.

Patient 6

The first child of consanguineous Iraqi parents presented to local hospital with poor feeding and lethargy on day 3. Feeds were stopped and the child treated empirically for septicaemia, with intravenous antibiotics and 10% dextrose-containing fluids. The newborn screening test, taken on day 3 and reported on day 8, was indicative of HMGCL deficiency. The baby was transferred to a tertiary unit and commenced on a protein- and fat-restricted diet, comprising 100mL/kg/day of breast feeds and 50mL/kg/day of maltodextrin solution supplemented with micronutrients. S-DL-3OHB was prescribed (150mg/kg twice daily). Solids were introduced at five months, with no high protein foods and fat limited to <3g fat per 100g, in addition to breastfeeding. By 12 months she was on approximately five bottles of 180mL of standard infant formula preceded by 35mls of maltodextrin solution. She had a maximum of two small serves of high protein foods per day.

At 18 months protein (from low fat sources) had increased to 3.4g/kg (16% of total energy) with fat contributing 20% energy and a maximum fasting interval of eight hours. Uncooked cornstarch 2g/kg nocte was introduced at this stage. Ketone dosing was rationalised to nocturnal treatment only.

At three years of age after a busy day at a wedding, this patient went to sleep omitting dinner, nocturnal UCCS and ketones. She had a hypoglycaemic fit early the next morning. This responded to 24 hours of intravenous dextrose and 4hrly ketones, and she was discharged on regular management. Fat intake remains restricted (as low as 8% of total energy at times); she has maintained a protein intake of ~3.4g/kg and continues to take nocturnal cornstarch and ketones. Emergency management for illness includes frequent feeds of maltodextrin solution to meet 120% of her EER. She is now seven years of age.

Patient 7

Patient 7 is the first child to first cousin consanguineous parents of Lebanese background. He was referred at two years of age, when he was noted to have persistent 3-methylglutaconic aciduria on two urine metabolic screens performed in the context of mild expressive and receptive language delay.

He was born at 38 weeks, with no concerns in the antenatal or early postnatal period. He navigated early illnesses without much difficulty, including an emergency department presentation with viral gastroenteritis at two years of age that did not require any intravenous fluid therapy. He had a normal audiology assessment and had already been receiving intensive early intervention with speech therapy, psychology and occupational therapy.

Urine organic acids demonstrated gross increase in 3-MGC, with a slight increase in 3-HMG and 3-MGL. Plasma acylcarnitine profile demonstrated a mild increase in methylglutarylcarnitine (0.12umol/L, upper limit of normal 0.08). Molecular testing of a panel of genes associated with 3-methylglutaconic aciduria identified a homozygous stop-loss variant in the *HMGCL* gene (c.975_976del, causing p.Ser326ext*5).

He is now three and half years old: from a developmental perspective, he has good gross motor and fine motor function, but continues to have issues with speech and language. Formal assessment for autism spectrum disorder is pending. He is growing well, with a most recent weight on the 55th centile and height on the 62nd centile. He has an unwell management plan that involves frequent

carbohydrate intake. He does not have a natural protein restriction; he is quite fussy but does have serves of meat and dairy. Interestingly, despite this diagnosis he has not had any significant metabolic decompensations to-date.

Patient 8

Patient 8 is a two year-old female; she is the first born child to a consanguineous couple of Pakistani ethnicity. She presented on day 6 of life with 14% weight loss and metabolic acidosis. Venous blood gas showed pH 7.16, pCO2 34 mmHg (41-51), bicarbonate 12 mmol/L, base excess -15.2 mmol/L, and glucose 3.5 mmol/L. She was commenced on intravenous bicarbonate and nasogastric expressed breastmilk. Newborn screening demonstrated a raised C5OH level, and urine organic acids at the time of the presentation demonstrated significantly raised 3-MGC and 3-HMG, in a pattern consistent with HMG-CoA lyase deficiency. The diagnosis was later confirmed on molecular testing. Following diagnosis carnitine supplementation was commenced. She was not placed on any dietary restrictions.

The patient has remained clinically well since diagnosis. She had two admissions following diagnosis with raised ammonia at two and three months of age but was clinically well on both occasions. During the admissions she was managed with nasogastric formula supplementation. During the second admission 8% maltodextrin was introduced into her feeds, which was reduced to 4% on discharge. Ketone supplementation was introduced at this time at a dose of 150mg/kg/day. She has a mild developmental delay and her parents have been slow with the introduction of solids into her diet. She remains on carnitine and ketone supplementation, without any dietary protein or fat restriction. Uncooked cornstarch at 1/g/kg/day has recently been introduced into her night-time feed.

Patient 9

Patient 9 is an 18 month old male; the fourth child to a non-consanguineous Caucasian couple. He was born at term at 2.81kg and had a normal newborn screen on day 2 of life. He had normal development and growth, and was predominantly formula fed, with slow progress with solids. He presented at nine months of age with acute liver failure and encephalopathy, in the context of 24-48 hour history of upper respiratory tract infection, for which he was treated at home with oral paracetamol and chlorine salicylate oral teething gel. Ambulance officers attended the home due to acute loss of consciousness and recorded a GCS of 10/15 (E4, V1, M5), blood glucose of 7.9 mmol/L and blood ketones of 0.6 mmol/L. He had a core body temperature of 35.7 degrees Celsius, bradycardia (68 beats per minute) and hypertension 140/80 mmHg. Hospital assessment additionally identified mild palpable hepatomegaly.

Venous blood gas showed pH 7.31, pCO2 29 mmHg (40-50), bicarbonate 15 mmol/L (22-32), base excess -10mmol/L (-3 to +3), glucose 8.1 mmol/L and lactate 2.6 mmol/L. Liver function tests were deranged, with AST 2450 U/L (20-110), ALT 1940 U/L (10-50), ammonia 62 mmol/L (<50), INR 2.6 (0.8-1.1) and prothrombin time 30.2 sec (9.8-13.0). A paracetamol level was 36 mg/L (critical level at four hours <150mg/L). The patient remained bradycardic and encephalopathic, and was given a single dose of intravenous vitamin K, commenced on an N-acetylcysteine infusion along with 5% dextrose and 0.9% sodium chloride infusion, and transferred to a tertiary paediatric centre.

Following transfer to a tertiary paediatric centre, the patient had further deterioration in liver function and coagulopathy. He had a single episode of hypoglycaemia at 2.2 mmol/L (3.5-5.5). He remained

significantly encephalopathic and was transferred to paediatric intensive care, where he continued on a 5% dextrose infusion. The transaminitis and coagulopathy peaked on day 3 of admission: AST 21,992 U/L (20-110), ALT 18,213 U/L (10-50), INR 9.8 (0.8-1.1) and prothrombin time 116.1 sec (9.8-13.0); the lactate peaked at 7.2 mmol/L. Rhinovirus was identified on respiratory virus PCR. The metabolic service was consulted on day 3 of the admission due to concern with the patient's persistent and unusual level of encephalopathy, despite correction of hypoglycaemia and hyperammonaemia for 48 hours.

Urine organics completed at admission identified gross increase in 3-MGC, and moderate increases in 3-HMG and 3-hydroxyisovalerate highly suggestive of HMGCL deficiency. Interestingly there were also significant elevations in 3-hydroxybutyrate, hexanedioate, octanedioate, 3-hdryoxydecanedioate, and 3-hydroxydodecaedioate consistent with severe ketosis. These findings were confirmed on a second sample 12 hours later. Acylcarnitines were collected on day 4 of admission and were unremarkable but for a significantly reduced free carnitine (2 umol/L; reference range 13-56) and total carnitine (8 umol/L; reference range 21-70).

Patient 9 was commenced on 900mg/kg/day S-DL-3OHB in six divided doses and 100mg/kg/day L-carnitine in four divided doses, and was switched to a 10% dextrose-containing solution. Prior to definitive treatment, he had been drowsy, agitated but rousable; within 12 hours of commencement of therapy, he was sitting up in bed, smiling and requesting a bottle which he was able to feed from independently. Urine organics and plasma acylcarnitines completed 48 hours after commencement of therapy were normal. Treatment initiation is temporally indicated by the red arrow in the graph below.

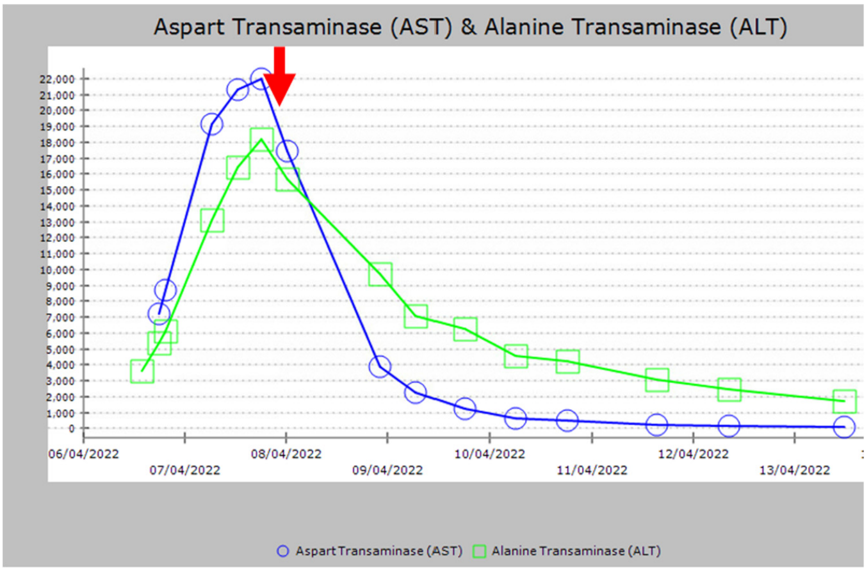

Formula feeds were modified with additional carbohydrate, micronutrient supplementation and skim milk to provide 1.7g/kg protein and 30% energy from fat, with a maximum fasting interval of six hours. 100mg/kg/day L-carnitine is continuing, and S-DL-3OHB was reduced to 200mg/kg/day in two divided doses. Intake of age-appropriate solid foods (<3g fat per 100g and no high protein foods) is increasing and nocturnal uncooked cornstarch (0.7g/kg) has been introduced to develop tolerance. Emergency management for illness includes frequent feeds of maltodextrin solution to meet 120% of his EER.

He had a second acute admission four weeks following the initial diagnosis, in the setting of COVID-19 illness. He again experienced rapid deterioration in level of consciousness and interactions, despite no interruption of his normal feeds. He was commenced on rescue S-DL-3OHB doses at home and was alert by the time he arrived at the emergency department. His liver function had normalised prior to this presentation and remained normal throughout the admission. Acylcarnitines remained normal.

His parents have commented since discharge from his initial presentation that he is more alert and interactive than previously noted, and had a 'developmental leap', with improved speech and language skills (two words to 5-6 words within 1 week). They also report that his is more active throughout the day.

Patient 10

Patient 10 is a nine month-old female, the fourth child to a consanguineous couple. She was born at term at 2.72kg and had newborn screening on day 1 of life. This identified an elevated C5OH of 1.75 which persisted on repeat testing on day 11 of life (1.99); her parents were contacted for urgent review on day 13 of life. She was reported to be well at home. She had regained her birthweight (3.02 kg) and was reported to feed well, though experienced small vomits/posits with alternate feeds. Examination was unremarkable.

Urine organics demonstrated gross increases in 3-MGC and 3-HMG, a moderate increase in 3-MGL and slight increase in 3-hydroxyisovalerate. Plasma acylcarnitines identified elevated 3-hydroxyisovalerylcarnitine 1.26 umol/L (<0.1), and methylglutarylcarnitine 1.2 umol/L (<0.08), biochemically confirming HMGCL deficiency. Dried blood spot biotinidase activity was normal.

She was commenced on 100mg/kg/day L-carnitine in two divided doses and 100mg/kg/day S-DL-3OHB also in two divided doses, along with modified feeding (100ml/kg standard infant formula + 50ml/kg maltodextrin solution + micronutrient supplement). Emergency management for illness includes frequent feeds of maltodextrin solution to meet 120% of her EER. Her mother reported that her vomiting resolved within 48 hours and she was more interactive at home. She has remained well and is growing and developing normally. She is able to sit unsupported, crawl and has polysyllabic babble. Apart from her initial mild clinical presentation, she has not had any episodes of acute metabolic decompensation.
